# Master protocol trials in oncology: Review and new trial designs

**DOI:** 10.1016/j.conctc.2018.08.009

**Published:** 2018-08-24

**Authors:** Akihiro Hirakawa, Junichi Asano, Hiroyuki Sato, Satoshi Teramukai

**Affiliations:** a Department of Biostatistics and Bioinformatics, Graduate School of Medicine, The University of Tokyo, Tokyo, 113-8654, Japan; b Biostatistics Group, Center for Product Evaluation, Pharmaceuticals and Medical Devices Agency, Tokyo, 100-0013, Japan; c Department of Biostatistics, Graduate School of Medical Science, Kyoto Prefectural University of Medicine, Kyoto, 602-8566, Japan

**Keywords:** Master protocol, Basket, Umbrella, Platform, Bayesian adaptive method

## Abstract

In oncology, next generation sequencing and comprehensive genomic profiling have enabled the detailed classification of tumors using molecular biology. However, it is unrealistic to conduct phase I–III trials according to each sub-population based on patient molecular subtypes. Common protocols that assess the combination of several molecular markers and their targeted therapies by means of multiple sub-studies are required. These protocols are called “master protocols,” and are drawing attention as a next-generation clinical trial design. Recently, several reviews of clinical trials based on the master protocol design have been published, but their definitions of these such trials, including basket, umbrella, and platform trials, were not consistent. Concurrently, the acceleration of the development of new statistical designs for master protocol trials has been underway. This article provides an overview of recent reviews for master protocols, including their statistical design methodologies in Oncology. We also introduce several examples of previous and on-going master protocol trials along with their classifications by some recent studies.

## 1 Introduction

In oncology, next generation sequencing and comprehensive genomic profiling have enabled detailed classification of tumors using molecular biology. With this development, targeted therapies are being established for some tumor types based on genetic mutations [[1], [2], [3], [4], [5], [6], [7], [8], [9]]. If patient groups of the same tumor type (for example, gastric, lung, breast, or colorectal cancer) are classified by molecular subtypes, such as by genetic mutation, then patient groups can be further subdivided into unique subgroups. However, it is unrealistic to conduct phase I–III trials according to each subpopulation [[10], [11], [12]]. Common protocols that assess the combination of several molecular markers and their targeted therapies by means of multiple sub-studies are required for single and/or multiple tumor types. These protocols are called “master protocols,” and are drawing attention as a next-generation clinical trial design.

Three challenges that have been particularly difficult in common clinical trials are possibly alleviated by conducting multiple clinical trials based on a master protocol [13]. First, inter-patient and intra-patient heterogeneity can be evaluated efficiently [14]. Identical tumor types can exhibit different responses to treatments depending on patient characteristics or disease stage, and even within the same patient, differences in the type of cancer cells within the tumor tissue can also generate a different treatment response. In trials using a master protocol, trial data from multiple sub-studies can be comprehensively used to evaluate inter- and intra-patient heterogeneity. Second, findings on specific signal pathways strongly associated with driver gene mutations and cancer cell growth and progression can be obtained [[15], [16], [17]]. Third, combining two or more targeted therapies makes it possible to expand the genetic mutations being studied [[18], [19], [20]].

This article begins with an overview of the history from biomarker-based trial design to master protocol trial, and subsequently summarizes clinical trials using master protocols in oncology based on recent general theories of master protocol design [[21], [22], [23]]. We introduce several examples of master protocol trials along with their classifications according to previous studies. We also discuss new statistical designs for basket trials, including designs that use the recently developed response-adaptive randomization, in addition to discussing the future direction of master protocol trials.

## 2 Changes from biomarker-based to master protocol trial design

In order to understand the motivation and concept of clinical trial design using a master protocol, we first introduce the clinical trial designs that use molecular markers, along with their history in cancer treatment. In oncology, patients are generally classified by their primary cancer and stage, and randomized controlled trials are conducted for each patient population to create standard therapies. Historically, cytotoxic agents have been develop based on this perspective. However, research and development in the 2000s enabled cancer cell growth and progression to be defined at cellular and molecular levels, and the presence or absence of molecular markers or genetic mutations enabled classification of particular tumor types into several subtypes. At the same time, there were developments in the chemotherapeutic drugs available, shifting from treatments centered on cytotoxic agents to those using molecularly targeted agents, which act selectively on cancer cells. Recently, there is active research on immune checkpoint inhibitors, which attack cancer cells by utilizing a patient's immune response. Molecularly targeted agents that target specific molecular markers include gefitinib and erlotinib for *EGFR* gene mutation-positive inoperable, recurrent, or metastatic non-small cell lung cancer [5,24], as well as crizotinib, alectinib, and ceritinib for *ALK* fusion gene-positive non-small cell lung cancer [[6], [7], [8],^25^]. Immune checkpoint inhibitors presently include nivolumab and pembrolizumab [26,27].

Clinical trial designs based on molecular markers began to gain popularity with the aforementioned changes in chemotherapy agents. Trial designs called “enrichment designs” or “targeted designs” are studies where patient populations with a single molecular marker for which a drug's effects can be expected in a specific tumor type (in this paper, we will assume that it is effective for the marker-positive population). This design is selected on the premise that: i) the molecular marker is an established marker, which is strongly correlated with the efficacy of an investigational drug; ii) it has been biologically demonstrated that drug efficacy cannot be expected in the marker-negative cases; and iii) that a diagnostic tool for evaluating molecular marker status has been developed. Clinical trials using the enrichment design have included a clinical trial of trastuzumab [1], the N9831 trial for *HER2*-positive breast cancer [28], and the ToGA trial on *HER2*-positive stomach cancer [11]. If the molecular marker is not established as a reliable marker, the use of a marker-stratified design may be considered. In this design, patients were assigned to arms by molecular marker positivity or negativity, and were randomized within each arm. Clinical trials that used the marker-stratified design include the INTEREST [29] and MARVEL [30] trials. After this type of design was introduced, sequential subgroup-specific, marker sequential test (MaST) and fallback designs were proposed as extensions of the marker-stratified design [31]; this eventually led to the proposal of clinical trials that use the master protocol design.

## 3 Master protocol trial

### 3.1 Definition and characteristics

**Fig. 1:**
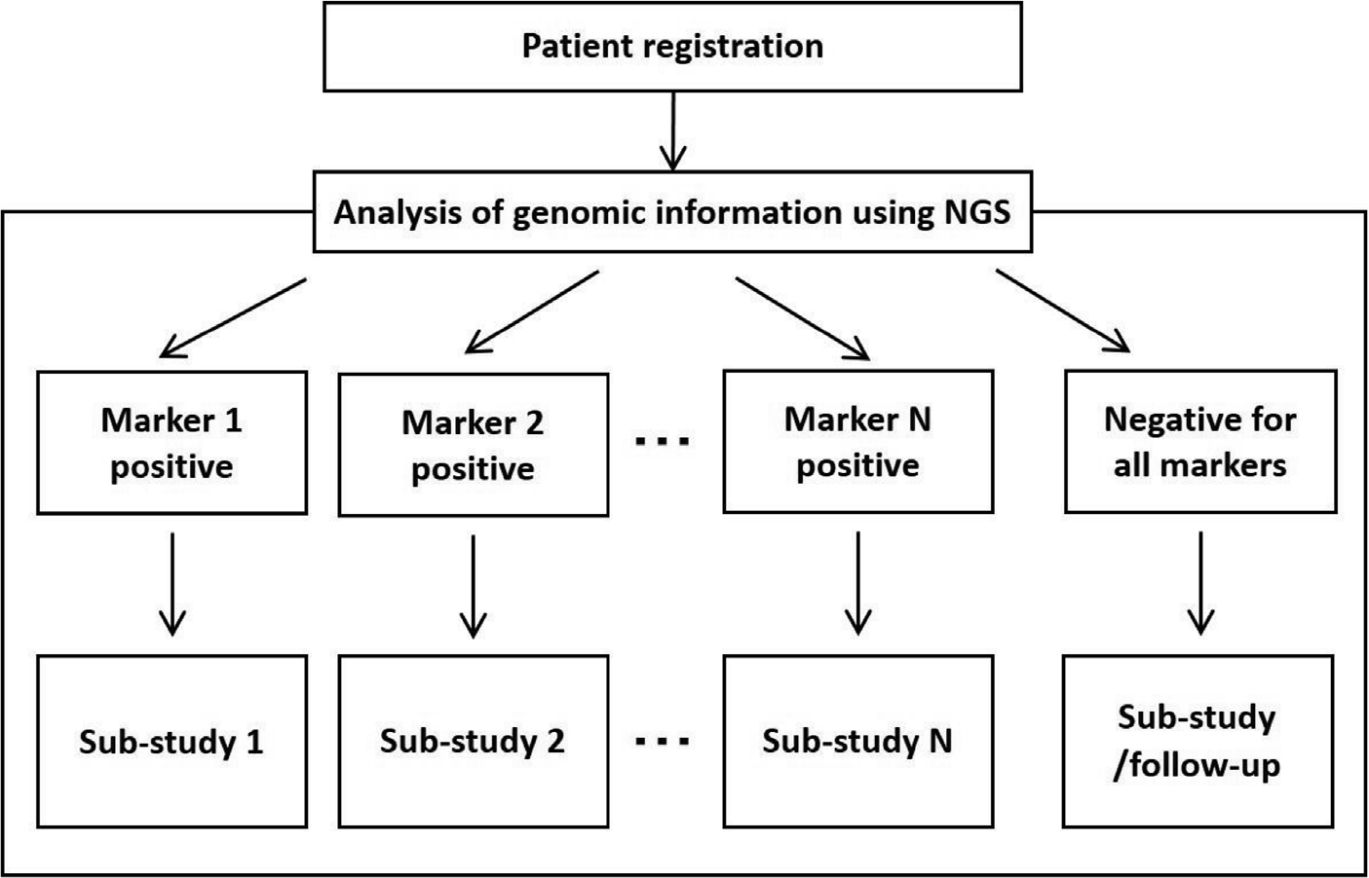
Master protocol trial.

A master protocol is a comprehensive protocol created for evaluating multiple hypotheses of sub-studies that are concurrently conducted. This comprehensive protocol comprises different sub-protocols of multiple concurrently-operating sub-studies (Fig. 1), where the sub-studies are commonly conducted on populations based on specific tumor types, histologic types, and/or molecular markers. We will refer to these types of trials as “master protocols.”

A master protocol trial uses a common system for patient selection, logistics, templates, and data management [22]. Histologic and hematologic specimens of patients enrolled in master protocol trials are also measured and analyzed using a common basic system (e.g., next generating sequencer and immunohistochemistry) to collect coherent molecular marker data. Patients can participate in sub-studies for which they meet eligibility criteria based on their molecular marker data. Thus, enrolling in a master protocol trial increases the chance of participation in a trial that is most suitable for a given patient. Even if there are no sub-studies that a given patient can participate in, they will be followed-up, and can be placed on a waiting list until an appropriate sub-study is started. Furthermore, natural history data from a waiting-list can be used as controls in evaluating the efficacy of an investigational drug in a single-arm sub-study.

### 3.2 Trial purpose

Master protocol trials can be exploratory or confirmatory [10,[32], [33], [34]]. Exploratory master protocol trials are often composed of multiple single-arm sub-studies, and confirmatory master protocol trials are composed of multiple randomized sub-studies. For either trial type, the design and statistical considerations are commonly standardized between all sub-studies.

### 3.3 Advantages and challenges

The advantages of a master protocol trial are related to the fact that they include data from sub-populations on a broad range of molecular markers. In comparison with the marker-based trials described in Section 2, two advantages appear in the master protocol trials. First, this enables efficient enrollment of rare fraction patients so that centralized patient management, based on a common protocol, promotes the acceleration of clinical development. Second, master protocol trials are beneficial for patients as well because they increase the chance of trial participation for which they can expect optimal therapeutic effects. On the other hand, the challenges associated with master protocol trials, include the fact that several small sub-studies are being conducted in parallel, which may increase the rate of false positive findings.

## 4 Basket, umbrella, and platform trials

### 4.1 Definitions

A master protocol trial is often classified into basket, umbrella, and platform trials based on characteristics of the study population (e.g., disease, histologic type, molecular marker) and on both the type and number of study therapies. The common definitions of each trial type based on literature [22,23] are shown in Table 1. However, as pointed out by Renfro and Sargent [23], the definitions of each trial type are not standardized [10,20,[35], [36], [37]], with possible overlaps between them that should be noted. For example, the NCI-MATCH trial, which will be mentioned in a later section, is a type that has aspects of both basket and umbrella trials. In this article, we will organize the trial types by definitions given in recent works [[21], [22], [23]].

**Table 1:** Common definitions of each trial type.

Trial type	Common definition in literature [22,23]
Basket	Evaluate one targeted therapy on multiple diseases or multiple disease subtypes.
Umbrella	Evaluate multiple targeted therapies for at least one disease.
Platform	Evaluate several targeted therapies for one diseases perpetually, and further accept additions or exclusions of new therapies during the trial.

### 4.2 Basket trials

**Fig. 2:**
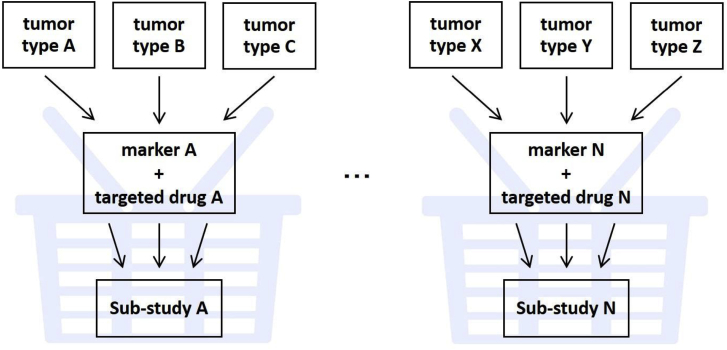
Basket trials.

A basket trial evaluates one targeted therapy on multiple diseases or multiple disease subtypes. In oncology, this is exemplified by examining the therapeutic effects of molecularly targeted agents for several tumor types that may have a common single molecular marker, or genetic mutation, by tumor type and/or across tumor types (Fig. 2). In this scenario, the grouped tumor types form a basket, and sub-studies are conducted by tumor groups within it. Basket trials are often conducted as single-arm, phase II trials with the purpose of evaluating proof-of-concept (POC) in an early stage of development. Generally, the number of participants in individual sub-studies are between 20 and 50, and hypotheses that can demonstrate statistical significance are made only when there is major therapeutic efficacy; therefore, a basket trial is considered a “signal-finding” trial. As for sub-study design, two-stage or multi-stage designs may be used. As such, basket trials are characterized by the comprehensive execution of single-arm trials with a small number of patients, which enables efficient patient enrollment for rare cancers or rare fractions. However, it should be noted that basket trials have the assumption that they allow a fairly accurate prediction of whether a tumor with particular molecular characteristics will respond to a targeted therapy; furthermore, such response to a targeted therapy is established irrespective of the histologic type of the tumor. Moreover, patients enrolled in each sub-study are often composed of a heterogeneous group in terms of tumor type, histologic type or patient characteristics. Therefore, as it is difficult to evaluate time-to-event endpoints (e.g., progression-free survival or overall survival), primary endpoints are often response rates, which are less sensitive to the effects of population heterogeneity. The absence of a control group is a limitation in evaluating therapeutic effect; thus, it is desired to collect control data.

### 4.3 Umbrella trials

**Fig. 3:**
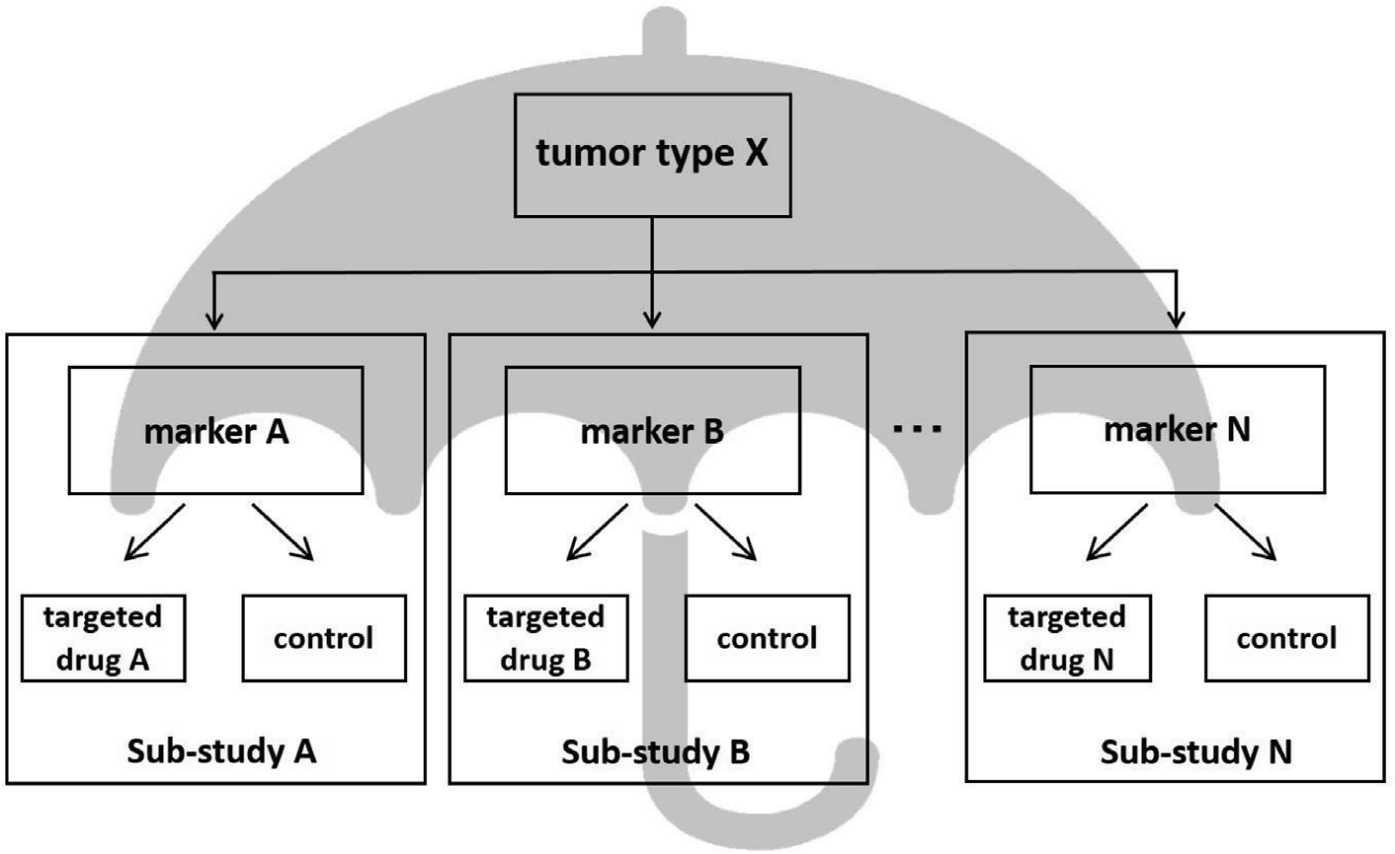
Umbrella trials.

Umbrella trials evaluate multiple targeted therapies for one disease or several diseases (e.g., that are expected to respond to an investigational drug). In oncology, sub-studies are conducted to evaluate targeted therapies that correspond to different molecular markers or genetic mutations within a particular tumor type. In that case, the tumor type is the “umbrella,” under which sub-studies for each molecular marker are operated (Fig. 3). Sub-studies may be single arm, phase II, or phase II/III trials that are randomized and compared to placebo or a standard therapy. Umbrella trials have in common a system that unifies molecular profiles of patient specimens for evaluation. While basket trials are generally single-arm sub-studies that are exploratory in nature, umbrella trials are often single-arm or randomized sub-studies that are confirmatory. Therefore, a randomized sub-study with appropriate eligibility criteria by tumor type and/or stage can generate confirmatory evidence related to the targeted therapy for the tumor type under study. However, patient enrollment may be slowed in the case of compartmentalization by molecular markers for examining rare cancers or rare fractions. In addition, umbrella trials are normally large-scale and long-term master protocol trials, but when the standard therapy changes during that period, the clinical significance of comparing to a control group of patients undergoing standard therapy is lost [10,20]. Hyman et al. [35] have termed umbrella trials as “molecular allocation studies.”

### 4.4 Platform trials

Platform trials evaluate several targeted therapies for one diseases perpetually, and further accept additions or exclusions of new therapies or patient populations during the trial. Basket and umbrella trials could also be considered platform trials, if they permit the addition or exclusion of new treatments during the trial. In a platform trial, interim analyses evaluate the efficacy or futility of each targeted therapy, and their results are used to exclude certain targeted therapies or to add new ones. Futility is often evaluated by the Bayesian method [33,35,38]. Since sub-studies by molecular markers are not mutually independent trials, the efficacy of the targeted therapy of each sub-study can be estimated by a Bayesian hierarchical model [23]. As such, platform trials permit relatively flexible addition or exclusion of treatment methods or patient populations, thereby enabling an efficient transition to a confirmatory trial. Challenges of the platform trial include its large-scale, long-term nature, associated high costs of managing and executing the trial, and the need to build organizations or frameworks that can operate these trials perpetually.

## 5 Example trials

Table 2 lists examples of basket trials, umbrella trials, and platform trials found in the literature. As mentioned above, the distinctions between each are not clear-cut; therefore, it should be noted that different authors may categorize these trials differently. An overview of several of these trials follows.

**Table 2:** Examples of master protocol trials in oncology.

Trial name	Design	Targeted drug	Main diseases	target	Number of patients	Endpoint	Woodcock & LaVange [22]	Cunanan et al. [21]	Renfro & Sargent [23]	Renfro & Mandrekar [13]
B2225	Ph2 (phase 2), multi-center, unblinded, uncontrolled	Imatinib	40 carcinomas (solid, hematologic)	–	186	Response rate	basket	–	–	–
BRAF V600	Ph2, multi-center, unblinded, uncontrolled	Vemurafenib, cetuximab	Multiple carcinomas	BRAF V600	122	Response rate	basket	basket	–	–
AcSe	Ph2, multi-center, unblinded, uncontrolled	Crizotinib	Gastrointestinal, breast, cancer, ovarian, thyroid cancers, sarcoma, etc.	ALK, MET, RON, ROS1	488	Response rate	–	–	basket	–
NCI-Match	Ph2, multi-center, uncontrolled	30 approved and unapproved treatments targeting genetic mutations	Advanced solid tumors, lymphomas, myelomas (mutation measured)	EGFR, MET, ALK, ROS1, BRAF V600, mTOR, etc.	35/sub-study	Response rate, PFS rate	umbrella	–	basket	basket
ALCHEMIST	Ph3, multi-center, unblinded, randomized	Erlotinib, crizotinib, nivolumab	Pulmonary adenocarcinoma	EGFR, ALK, PD-L1	≥770	OS, DFS	–	–	umbrella	umbrella
LUNG-MAP	Seamless Ph2/3, randomized	Erlotinib, ipilimumab, nivolumab, palbociclib, etc.	Squamous cell non-small cell lung carcinoma (NSCLC)	PIK3CA, FGFR, CCDN1, D2, CDK4, c-MET	100-170 (Ph2) 300-400 (Ph3)	Response rate, PFS, OS	master protocol	–	umbrella	
FOCUS4	Ph2/3, multi-center, unblinded, randomized	Molecular target drugs such as BRAF inhibitors, etc.	Colorectal cancer	BRAF, PIK3CA, PTEN, KRAS, NRAS	Approx.2000	PFS, OS	–	–	umbrella	platform
BATTLE-1	Ph2, single-center, adaptive-randomized	Erlotinib, vandetanib, sorafenib, erlotinib + bexarotene	Advanced NSCLC	EGFR, KRAS, BRAF, VEGF, RXRs/CyclinD1	255	Response rate, PFS, OS, toxicity	umbrella	–	platform	–
I-SPY 2	Ph2, multi-center, adaptive-randomized	Pembrolizumab, trastuzumab, pertuzumab, neratinib, etc.	Early high-risk breast cancer	HR, HER2, MammaPrint	1920	Pathological response rate	platform	–	platform	–
SHIVA	Ph2, multi-center, unblinded, randomized	Erlotinib, lapatinib + trastuzumab, sorafenib, imatinib, dasatinib, vemurafenib, everolimus, abiraterone, letrozole, tamoxifen	Advanced tumors resistant to standard treatment	Hormone receptor, PI3KA, KT, mTOR, RAF, MEK	195	PFS	–	–	platform, etc.	–
NCI-MPACT	Ph2, multi-center, unblinded, randomized	PARP inhibitors, Wee 1 inhibitors, everolimus, trametinib	Advanced solid tumors	DNA repair, PI3K, RAS/RAF/MEK	180	Response rate, PFS rate	–	–	platform, etc.	–
CUSTOM	Multi-center, unblinded, uncontrolled	Erlotinib, selumetinib, AKT inhibitors, lapatinib, sunitinib	NSCLC, small cell lung carcinoma, thymic carcinoma	EGFR, KRAS, HRAS, NRAS, BRAF, PTEN, Akt 1, PIK3CA, ERBB2, KIT, PDGFRA	647	Response rate	–	basket	platform, etc.	–
CREATE	Ph2, multi-center, unblinded, uncontrolled	Crizotinib	Advanced cancers such as sarcomas	ALK, MET	582	Tumor regression	–	basket	platform, etc.	–

OS: Overall survival, PFS: Progression-free survival, DFS: Disease-free survival.

### 5.1 Study B2225

Study B2225 enrolled patients with 40 different solid tumors or hematologic malignancies, without limiting molecular markers to evaluate the efficacy of imatinib [39]. The sample size for each tumor type was not determined in advance, but patients were enrolled competitively. Although it did not begin as a basket trial, it is categorized as a basket trial by Woodcock and LaVange [22] because it studied multiple tumor types for one drug.

### 5.2 BRAF-V600 study

The BRAF-V600 study is a trial comprising multiple sub-studies to evaluate the efficacy of vemurafenib for non-melanoma *BRAF* V600E mutation-positive cancers [40]. Hyman et al. [40], Cunanan et al. [21], and Woodcock and LaVange [22] all classified this study as a basket trial. The sample size for each sub-study is determined by the Simon two-stage design.

### 5.3 AcSé trial

The AcSé (Accès Sécurisé à des thérapies ciblées innovantes) is an ongoing trial composed of 23 sub-studies that evaluate the efficacy of crizotinib monotherapy for several tumor types associated with *ALK*, *MET*, *RON*, and *ROS1* mutations [20]. Menis et al. [20] reviewed the master protocol trial on thoracic malignancies and categorized the AcSé trial as a basket trial. The associated sub-studies were typically based on a two-stage design.

### 5.4 NCI-MATCH trial

The NCI-MATCH (NCI Molecular Analysis for Therapeutic Choice) trial is composed of 24 sub-studies that evaluate the efficacy of at least 17 targeted therapies for solid tumors and lymphomas that were treated with at least one regimen [41]. The primary endpoint for each sub-study is response rate. Thirty-five patients were enrolled in this single-arm design trial, based on a binomial distribution, and the key secondary endpoint was the 6-month progression-free survival. Woodcook and LaVange [22] categorized the NCI-MATCH trial as an umbrella trial, but Renfro and Sargent [23] categorized it as a basket trial.

### 5.5 ALCHEMIST trial

ALCHEMIST (The Adjuvant Lung Cancer Enrichment Marker Identification and Sequencing Trial) trial is a randomized umbrella trial on *ALK*- or *EGFR*-positive, high-risk lung adenocarcinoma patients [42]. *ALK*- or *EGFR*-positive patients are enrolled in a phase III randomized sub-study comparing crizotinib to placebo, or a randomized sub-study comparing erlotinib to placebo. The primary endpoint of each trial is overall survival and an interim analysis is planned. In patients who are both *ALK*- and *EGFR*-negative, PD-L1 expression is measured and they are considered for enrollment in a randomized sub-study that compares nivolumab administration group to an observation group. The primary endpoints for these sub-studies are overall survival and disease-free survival. Similar umbrella trials include the LUNG-MAP trial [23].

### 5.6 FOCUS4 trial

The FOCUS4 trial is a placebo-controlled multi-group multi-stage randomized trial to evaluate the efficacy of multiple targeted therapies for untreated colorectal cancer [43]. Safety is evaluated in stage 1, and POC is confirmed in stage 2, short-term outcomes evaluated in stage 3, and long-term efficacy is evaluated in stage 4 in a patient population with a particular molecular marker. The efficacy endpoints are progression-free survival and overall survival. A multi-group, multi-stage design like this trial allows for new treatments to be added during the trial and for exclusion of treatments that have been confirmed to be futile before entering stage 3 or 4, which corresponds to phase III. This is the reason Renfro and Sargent [23] categorize the FOCUS4 trial as an umbrella trial, whereas Renfro and Mandrekar [13] have noted that it can also be classified as a platform trial. Patients who are negative or wild-type for all molecular markers can also enroll in the FOCUS4 trial.

The BATTLE-1 [[44], [45], [46]], I-SPY 2 [47,48], SHIVA [49], and CUSTOM [50] trials are often referenced as examples of platform trials in reviews on master protocol trials.

## 6 New trial designs in master protocol trials

A basket trial may include a single-arm sub-study for several tumor types with the same molecular marker, with response rate as the primary endpoint for a given tumor type. Occasionally, an interim analysis during the trial will discover that a sub-study for a tumor type with an extremely low response rate should be terminated early due to a lack of therapeutic effect. In addition to evaluating the response rate of each sub-studies sometimes it is of interest to evaluate response rates across different tumor types. Recently, several authors developed a new methodology for the basket design to accommodate these requirements. In this section, we overview these methods along with the existing approaches that can be used for the design of basket trials.

Before the emergence of master protocol trials, some statistical designs for single-arm trials that included multiple sub-populations have been proposed. As frequentist designs, LeBlanc et al. [51] proposed the frequentist strategy, including both analyses for each stratum, as well as a combined analysis that borrows information from all trial patients. Regarding Bayesian designs, Thall et al. [52] and Berry et al. [53] proposed the use of Bayesian hierarchical modeling, which borrows information across sub-populations. For example, the basket trial (NCT02034110) investigating the efficacy of combined dabrafenib and trametinib therapy for rare cancers with BRAF-V600 gene mutations estimated the response rate using the Bayesian hierarchical model. However, Freidlin and Korn [54] reported that in the phase II setting, the hierarchical Bayesian approach did not work well in the simulations considered, as there appears to be insufficient information in the outcome data to determine whether borrowing across subgroups is appropriate. These approaches would be applied to the response rate obtained from basket sub-studies in basket trials. On the other hand, several authors have recently developed new statistical designs for master protocol. For example, Neuenschwander et al. [55] developed the exchangeability–nonexchangeability (EXNEX) method, which is a robust mixture extension of the standard EX approach and allows each stratum-specific parameter to be exchangeable with other similar strata parameters or nonexchangeable with any of the similar parameter. Simon et al. [56,57] developed a different kind of Bayesian design using two parameters, which one is specified as the prior probability that the response in each stratum is considered desirable activity and one is specified as the prior probability that the response probabilities are equal across strata. More recently, Chu and Yuan [58] developed the Bayesian latent subgroup design (BLAST), which accounts for patient heterogeneity. In frequentist approaches, Cunannan et al. [59] modified the method of using independent Simon two-stage designs for each basket to improve the overall efficiency of the trial overall. A hybrid design that combines the Bayesian and frequentist models (hybrid design) [60], has also been proposed. In this design, if the homogeneity of treatment effect among strata is not rejected at an interim analysis, the hierarchical Bayesian model is used at the final analysis. More recently, Chu and Yuan [61] developed a calibrated Bayesian hierarchical model approach to evaluate the treatment effect based on binary endpoint. Apart from these single-arm designs, several authors proposed other designs with different perspectives. For example, Yuan et al. [63] and Chen et al. [64] focused on the control of the family-wise error rate in basket trials with pruning. Response-adaptive randomization is also discussed in the literature [65,66].

In contrast to the basket trials, new designs for the umbrella and platform designs have been little studied. In the platform trials such as BATTLE and I-SPY2 trials, adaptive randomization and the Bayesian hierarchical models was used for evaluating the treatment effects [48,62,67]. Hobbs et al. [38] and Kaizer et al. [68] developed new Bayesian modeling and adaptive randomization for platform design. Ghosh et al. [69] developed the frequentist approach for platform trial, which guarantees strong control the type-1 error rate. Simon [62] reviewed some of the umbrella, basket, and platform designs and suggested using enrichment designs for phase II/III umbrella trials.

Response-adaptive randomization may also be used in the umbrella and platform trial types. However, it must be noted that there are many debates on response-adaptive randomization that are independent of the various problems associated with master protocol trials. Previous reports have discussed the designs of platform trials, including adaptive randomization [[70], [71], [72]].

## 7 Summary and prospects

The randomized controlled trial is the gold standard for establishing standard treatments. In principle, randomized controlled trials evaluate the safety and efficacy of one therapy for one disease, and are designed to evaluate a pre-established statistical hypothesis. However, the rising costs of operating clinical trials, including randomized controlled trials, have limited the feasibility of conducting randomized controlled trials for all important clinical hypotheses. To overcome this problem, new experimental designs and methods of data analysis have been developed to efficiently evaluate single or multiple hypotheses. For example, the group sequential design, sample size re-estimation, and adaptive designs, such as the seamless phase II/III design [73], can be considered as trial designs for evaluating hypotheses efficiently.

In oncology, new clinical trials that use basket, umbrella, platform, or other master protocols are expected to increase due to the focus on genomic medicine. Clinical trials using master protocols are increasing in the non-Oncology setting as well. These include the REMAP-CAP (Randomized, Embedded, Multifactorial Adaptive Platform Trial for Community-Acquired Pneumonia) trial [74] in the area of bacterial and viral diseases, the ALIC^4^E (Antivirals for influenza Like Illness? A rCt of Clinical and Cost effectiveness in primary CarE) trial [75] for influenza like illness, the PREVAIL II (the Partnership for Research on Ebola Virus in Liberia II) trial for Ebola [76], and the EPAD LCS (the European Prevention of Alzheimer's Dementia Longitudinal Cohort Study) [77]) and the DIAN-TU NexGen (the Dominantly Inherited Alzheimer Network Trials Unit Next Generation) trials [78] of mental and neurological diseases.

In recent years, there have been drugs for which efficacy was not observed in a broad study population, but they did demonstrate marked efficacy in specific patients. These patients are called “exceptional responders” [79], and new initiatives are in place to elucidate their molecular profiles. Master protocol trials can also be used to identify exceptional responders and are anticipated to become one of the standard clinical trial designs to promote individualized medical care.

## Funding

This work was partially supported by the Grant-in-Aid for Scientific Research [grant number 17K00045] from the Japan Society for the Promotion of Science.

## Appendix A Supplementary data

Data Profile Data Profile
